# Detection and diagnostic value of serum NSE and S100B protein levels in patients with seizures associated with mild gastroenteritis: A retrospective observational study

**DOI:** 10.1097/MD.0000000000023439

**Published:** 2020-11-25

**Authors:** Hui Chen, Yong Chen, Jian Min Zhong

**Affiliations:** Department of Neurology, Children's Hospital of Jiangxi Province, Nanchang, China.

**Keywords:** convulsions, febrile seizures, gastroenteritis, neuronal-specific enolase

## Abstract

Benign convulsions with mild gastroenteritis (CwG) and febrile seizures (FS) associated with mild gastroenteritis are 2 different diseases in the spectrum of seizures associated with mild gastroenteritis. However, specific and useful indicators for the identification of the 2 diseases are lacking. A retrospective analysis was performed to compare the serum neuronal-specific enolase (NSE) and S100B protein levels between patients with these 2 diseases to evaluate the value of NSE and S100B for differential diagnosis between these 2 diseases.

The clinical data and NSE and S100B protein levels of 81 children with seizure-associated mild gastroenteritis were collected. According to the axillary temperature at the time of convulsions, all patients were classified into an afebrile seizure (AFS) group, hereafter called the CwG group (n = 46), and a febrile seizure group (FS group, n = 35).

The serum NSE level was higher in the CwG group than in the FS group (14.046 (11.095, 19.266) pg/ml and 9.034 (7.158, 12.165) pg/ml, respectively, *P* < .001); however, the serum S100B protein levels in the CwG and the FS group were not significantly different (*P* > .05). Receiver operating characteristic (ROC) curve analysis showed that the area under the curve (AUC) for NSE was 0.806, *P* = .000, which was statistically significant. The Youden index was largest (0.605) for a serum NSE cut-off value of 10.460 pg/ml, which yielded a sensitivity and specificity of 89% and 71%, respectively, for prediction of a CwG diagnosis.

NSE may contribute to the differential diagnosis of CwG and FS associated with mild gastroenteritis.

## 1 Introduction

Gastroenteritis is the main cause of hospitalization for infants and children younger than 3 years old. The incidence of gastroenteritis in infants and young children is approximately 0.5-2 times per person per year, and rotavirus is the main pathogen.^[1]^ Febrile seizure (FS) or afebrile seizure (AFS), meningoencephalitis and encephalopathy are the most common complications outside the gastrointestinal tract.^[2]^

Mild gastroenteritis is characterized by an absence of moderate to severe dehydration, electrolyte imbalance and hypoglycemia during gastroenteritis. The occurrence of seizures associated with mild gastroenteritis has recently been increasingly reported.^[3–5]^ AFS associated with mild gastroenteritis commonly occurs in infants and young children, which is often called benign convulsions with mild gastroenteritis (CwG). CwG was first proposed by the Japanese scholar Morooka in 1982,^[6]^ and this disease frequently occurs in East Asia. The peak age of onset is 1 to 2 years, and these patients account for approximately 1% of all acute gastroenteritis cases.^[7]^ A previous study showed that CwG represented 2% of children with convulsive epileptic seizures presenting to a Pediatric Emergency Department.^[8]^ Currently, the pathogenic mechanism is unknown but has received increasing attention in recent years.^[7]^ CwG is a syndrome that occurs in previously healthy infants and young children with mild gastroenteritis associated with AFS and is diagnosed after the exclusion of meningitis, encephalitis, encephalopathy, moderate to severe dehydration, electrolyte imbalance or hypoglycemia. CwG usually has a benign prognosis.^[7,9]^ Neuroimaging studies of CwG usually did not reveal any abnormalities. However, a previous study reported MRI diffusion-weighted image findings of 2 patients who were clinically diagnosed with CwG. Diffusion-weighted image demonstrated a transient abnormality in the splenium of the corpus callosum. Frequent seizures might cause transient splenial abnormality in patients with CwG.^[10]^ Another study reported a case of benign infantile convulsions due to norovirus gastroenteritis. MRI of the case showed transitory signal abnormalities consistent with vasogenic edema in bi-parietal cortico-subcortical, and the cerebral MRI after 1 month was negative.^[11]^ These 2 studies showed that very few patients with CwG might have transient brain damage

In clinical practice, gastroenteritis is sometimes accompanied by fever. FS associated with mild gastroenteritis is another type of disease on the spectrum of seizures associated mild gastroenteritis and shares many clinical symptoms with CwG, such as prevalence in a specific age range and gastrointestinal symptoms. A previous study reported that FS accounted for 2.2% of 755 cases of mild rotavirus enteritis.^[5]^ Currently, identification of the 2 diseases is mainly based on the axillary temperature at the time of convulsion (whether it is greater than 38°C), but in many cases, parents often forget to take their childrens temperatures because of panic at the time of convulsion, which complicates the clinical diagnosis. Clinically, specific and useful indicators for the identification of the 2 diseases are lacking. Numerous studies have shown that neuronal-specific enolase (NSE) and S100B protein levels can be used as biochemical markers of brain injury.^[12–14]^ In China, NSE and S100B protein levels have been widely used as indicators of brain injury in clinical practice. Previous studies have shown that a cluster of several seizures during a single diarrhoeal episode is common in patients with CwG.^[7]^ Kang et al reported that there were 88.1% patients in the CwG group who had 2 or more seizures, ranging from 1 to 8 episodes. Meanwhile, there were 35.3% patients in the FS group who had multiple seizures ranging from 1 to 4 episodes. In the acute phase of gastroenteritis, CwG had more convulsive episodes than FS.^[5]^ Their study also showed that no statistically significant difference was observed between the 2 groups in terms of the duration of each seizure.^[5]^ Therefore, we speculated that CwG had more obvious brain damage and CwG might have a higher NSE and S100B protein levels. Therefore, this study retrospectively analyzed the serum NSE and S100B protein levels of infants with CwG and compared them with those of children with FS associated with mild gastroenteritis to investigate the difference in indicators of brain injury between the 2 diseases. This study aimed to evaluate the values of the serum NSE and S100B protein levels for the differential diagnosis between CwG and FS associated with mild gastroenteritis because relevant studies are lacking.

## 2 Subjects and methods

Children with seizures associated with mild gastroenteritis diagnosed by the Department of Neurology of Jiangxi Children's Hospital from January 2015 to January 2018 were enrolled in this study. The inclusion criteria for patients with seizures associated with mild gastroenteritis^[5]^ were as follows:

1. patients did not have moderate to severe dehydration, an electrolyte imbalance or hypoglycemia;
2. patients exhibited convulsions during gastroenteritis and
3. patients had undergone serum NSE and S100B protein detection.

The exclusion criteria were as follows:

1. patients did not undergo follow-up;
2. patients with developmental delay, encephalopathy, encephalitis, traumatic brain injury, or brain tumors in their medical history or during follow-up and
3. patients with a history of epilepsy at the beginning of the study.

This study was approved by the Medical Ethics Committee of Jiangxi Provincial Children's Hospital, and informed consent forms were signed by all of the subjects guardians.

The patients were divided into an AFS group and an FS group according to their axillary temperature at the onset of convulsions. The AFS group is hereafter referred to as the CwG group because the patients in the AFS group met the definition of CwG.^[15]^ FS was defined as axillary temperature ≥38°C at the onset of convulsions, whereas axillary temperature less than 38°C at the onset of convulsions was classified as an AFS. If fever occurred during the interictal period but not during the episode, patients were classified into the CwG group. If a patient had both FS and AFS in one course, the patient was classified into the FS group.

Clinical data were collected, including age, gender, gastroenteritis symptoms, convulsions (seizure type, number of convulsions and total convulsion duration = sum of the duration of each convulsion), routine stool examination and etiological results, lumbar puncture results, electroencephalography (EEG),cranial imaging and serum NSE and S100B protein levels.

Five milliliters of peripheral venous blood was collected at the time of admission, coagulated at room temperature for 30 minutes and centrifuged at 1000 r/minutes for 15 minutes; then, the serum was collected. Specimens were stored at 2 to 8°C. The protein levels of serum NSE and S100B were detected by chemiluminescence according to the instructions of the kit (Shenzhen new industry biomedical engineering Co., Ltd.).

Statistical analysis was performed using SPSS 19.0 statistical software. Measurement data were subjected to normal distribution and homogeneity of variance tests. Data that conformed to a normal distribution and had homogeneity of variance are expressed as the mean ± standard deviation. Comparisons between 2 groups were performed using a t test. Measurement data that did not conform to a normal distribution are expressed as the median (25th percentile, 75th percentile) (M (P25, P75)). The Mann–Whitney *U* test was used for comparisons between the 2 groups. The Chi-Squared test was used to evaluate the count data. Correlation analysis was performed using a spearman correlation analysis. If the analysis yielded significant results, the receiver operating characteristic (ROC) curve was plotted using sensitivity as the ordinate and 1-specificity as the abscissa. The predictive value was measured by the area under the ROC curve (AUC), and the critical value with the highest diagnostic accuracy (i.e., the largest Youden index) was determined based on the curve. A value of *P* < .05 was considered statistically significant.

## 3 Results

### 3.1 Comparison of clinical characteristics between the CwG and FS groups

**Figure 1:**
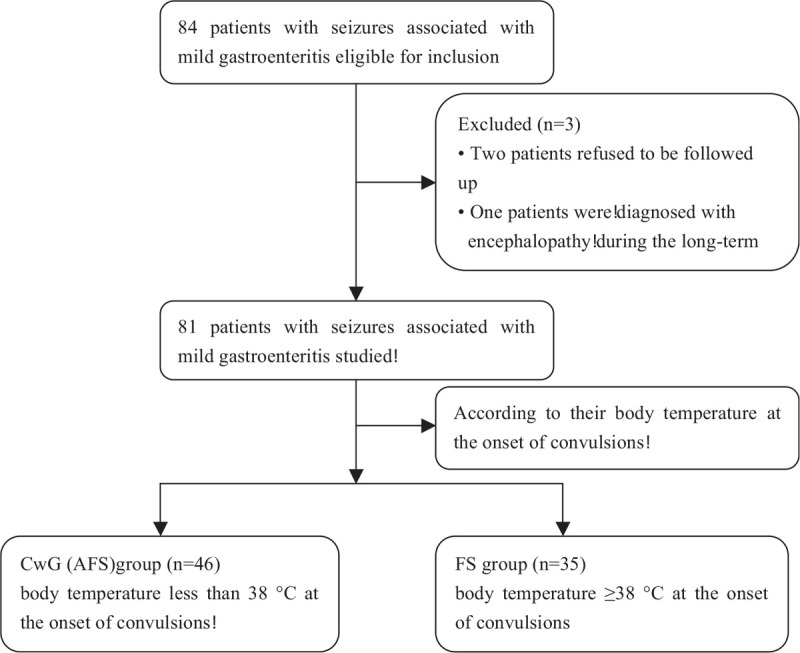
The study diagram for enrolled patients 46 patients in the CwG (AFS) group met the criteria, including 26 males and 20 females, and 35 patients in the FS group met the criteria, including 25 males and 10 females. AFS = afebrile seizure, CwG = convulsions with mild gastroenteritis, FS = febrile seizure.

After screening according to the inclusion criteria and exclusion criteria, 46 patients in the CwG group met the criteria, including 26 males and 20 females, with an average age of 17.80 ± 4.58 months, and 35 patients in the FS group met the criteria, including 25 males and 10 females (Fig. 1), with an average age of 16.49 ± 7.31 months. No significant difference was observed in gender composition or age between the 2 groups (*P* > .05). A total of 30 (65.22%) CwG patients had clustered seizures **(**≥2 convulsions**)** during the course of the disease, while only 4 (11.43%) patients in the FS group had clustered seizures, resulting in a statistically significant difference between the2 groups (*P* < .001). The CwG group had 108 episodes of convulsions, and 12 episodes (11.11%) were longer than 5 minutes. The FS group had a total of 39 episodes, and 18 (46.15%) episodes were longer than 5 minutes, which resulted in a statistically significant difference between the 2 groups (*P* < .001). Twenty four (52.17%) patients in the CwG group were positive for rotavirus, while 6 (17.14%) patients in the FS group were positive for rotavirus, resulting in a statistically significant difference between the 2 groups (*P* < .01). No statistically significant difference (*P* > .05) was observed between the 2 groups in terms of the type of convulsions and the total duration of convulsions (Table 1). According to the inclusion and exclusion criteria of this study, all subjects were required to have data of cranial imaging. Each of the included subjects had MRI data. All subjects had normal cranial imaging (MRI). None of the patients showed epileptic discharges on the interictal EEGs or had abnormal lumbar puncture results. However, In the CWG group, 35 (76.09%) patients had normal interictal EEG results and 11 (23.91%) had slow background. In the FS group, 28 (80.00%) patients had normal interictal EEG results and 7 (20.00%) had slow background. No statistically significant difference (*P* > .05) was observed between the 2 groups in terms of interictal EEG.

**Table 1:** Comparison of clinical characteristics and serum NSE and S100B levels between the CwG and FS groups.

	CwG (n = 46)	FS (n = 35)	*P* value
Age (months)	17.80 ± 4.58	16.49 ± 7.31	.35
Male gender	26 (56.52%)	25 (71.43%)	.17
Number of seizures			
Numbers of patients with 1 seizure	16 (34.78%)	31 (88.57%)	.00^∗^
Numbers of patients with ≥2 seizures	30 (65.22%)	4 (11.43%)	
Total convulsion duration (min)^a^	4 (2, 10)	5 (3,10)	.923
Duration of each convulsion			
<5 min	96 (88.89%)	21 (53.85%)	.00^∗^
≥5 min	12 (11.11%)	18 (46.15%)	
Seizure type			
Generalized seizure	97 (89.81%)	34 (87.18%)	.765
focal seizure	4 (3.70%)	2 (5.13%)	.656
unknown	7 (6.48%)	3 (7.69%)	.725
Rotavirus positive (case)	24 (52.17%)	6 (17.14%)	.001^∗^
NSE (pg/ml)	14.046 (11.095, 19.266)	9.034 (7.158, 12.165)	.00^∗^
S100B (ng/ml)	0.117 (0.082, 0.162)	0.109 (0.082, 0.266)	.60

All the patients in this study were followed-up closely for at least 24 months by telephone or face-to-face interview. All subjects had normal psychomotor development. In CwG group, 2 cases recurred in the form of CWG after 3 and 7 months, respectively. None of the cases eventually developed into epilepsy. However, in FS group, 16 cases recurred in the form of FS after the shortest interval of 3 months and the longest of 22 months, respectively. Five of the 16 cases eventually developed into epilepsy and received long-term antiepileptic treatment. The recurrence rates of the 2 groups were statistically significant (*P* < .05).

### 3.2 Comparison of serum NSE and S100B levels between the CwG and FS groups

The level of serum NSE in the CwG group was higher than that in the FS group (14.046 (11.095, 19.266) pg/ml and 9.034 (7.158, 12.165) pg/ml, respectively, *P* < .001), while the serum S100B protein levels in the CwG and FS groups were not significantly different (0.117 (0.082, 0.162) ng/ml and 0.109 (0.082, 0.266) ng/ml, respectively, *P* > .05, Table 1).

### 3.3 Optimal diagnostic cut-off value for predicting CwG by serum NSE levels

**Figure 2:**
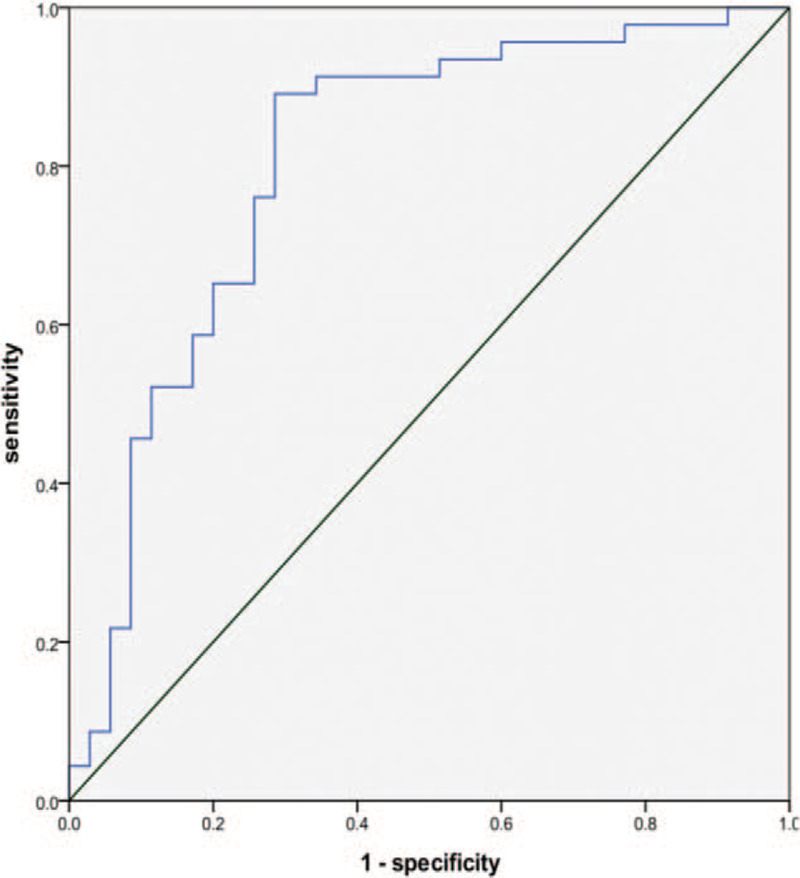
ROC curve of NSE in predicting a diagnosis of CwG. CwG = convulsions with mild gastroenteritis, NSE = neuronal-specific enolase, ROC = the receiver operating characteristic.

The serum NSE level was higher in the CwG group than in the FS group and was used as a predictor to determine whether the subjects would be diagnosed with CwG. A ROC curve was generated using SPSS statistical software (Fig. 2). The results showed that the AUC of the serum NSE level was 0.806, *P* = .000, which was statistically significant, indicating that the NSE level could predict a diagnosis of CwG. The Youden index was largest (0.605) for a serum NSE level of 10.460 pg/ml, and the sensitivity and specificity of the prediction of a CwG diagnosis using this value were 89% and 71%, respectively.

### 3.4 Correlation analysis of serum NSE levels, number of convulsions and total convulsion duration

The serum NSE level was not correlated with the number of convulsion or total convulsion duration in the CwG group (*P* > .05) or in the FS group (*P* > .05).(Table 2)

**Table 2:** Correlation analysis of serum NSE levels, number of convulsions and total convulsion duration.

	NSE levels of CwG	NSE levels of FS
Number of convulsions *r*^∗^	−0.011	−0.205
*P*	0.94	0.239
Total convulsion duration^†^*r*	−0.003	0.057
*P*	0.986	0.746

## 4 Discussion

This study summarized the clinical features of CwG and FS associated with mild gastroenteritis and retrospectively analyzed and compared the serum NSE and S100B protein levels of 46 patients with CwG and 35 patients with FS. The purpose of this study was to determine the value of serum NSE and S100B protein levels for the differential diagnosis between CwG and FS associated with mild gastroenteritis.

Many previous studies have shown that CwG occurs in infants and young children,^[16–18]^ and FS associated with mild gastroenteritis also occurs in infants and young children, with no significant difference in age between the CwG and FS groups.^[3–5]^ This study was consistent with previous reports. The average age of the CwG group was 17.80 ± 4.58 months, and the average age of the FS group was 16.49 ± 7.31 months, resulting in no statistically significant difference in age between the 2 groups (*P* > .05). In this study, 46 patients in the CwG group met criteria, including 26 males and 20 females, and 35 patients in the FS group, including 25 males and 10 females. There was no statistically significant difference in gender composition between the 2 groups (*P* > .05), which is also consistent with previous reports.^[3,4]^ However, Kang et al reported that their FS group consisted of 47% female patients, while the CwG group consisted of 64% female patients, resulting in a statistically significant difference between the 2 groups,^[5]^which may be related to selectivity bias.

Many previous reports have shown that CwG patients often have clustered seizures.^[19–23]^ Consistent with previous reports, 30 (65.22%) children with CwG in this study had clustered seizures, while the FS group had only 4 patients with clustered seizures (11.43%). The difference in clustered seizures between the 2 groups was significant (*P* < .001), indicating that pediatric patients with CwG are more prone to have clustered seizures than those with FS, which is consistent with previous reports.^[4]^ Lee et al reported that 3 of 27 children (11%) with CwG developed seizures lasting longer than 5 minutes, and up to 11 (34%) of the 32 children with FS had seizures longer than 5 minutes. The incidence of seizures in the FS group showed a trend of prolonged convulsions (more than 5 minutes), although no statistically significant difference was observed between the 2 groups (34% vs 11%, *P* = .063).^[3]^ This study further confirmed the results of our study. The present study included 108 episodes in the CwG group, and 12 episodes (11.11%) had a duration longer than 5 minutes. However, 39 seizure episodes occurred in the FS group, and 18 (46.15%) were longer than 5 minutes, resulting in a significant difference between the 2 groups (*P* < .001). In terms of the types of seizures, previous studies showed that the types of seizures in the CwG and FS groups mainly included generalized seizures.^[23]^ Consistent with previous studies, this study included 108 seizure episodes in the CwG group, and 97 (89.81%) were generalized seizures. The FS group included 39 seizure episodes, and 34 (87.18%) manifested as generalized seizures, resulting in no significant difference in the types of seizures between the 2 groups (*P* > .05). Higuchi et al reported that 21 of 76 (27.6%) patients with CwG were positive for rotavirus, while 18 of 50 (36.0%) patients in the FS group were positive for rotavirus, resulting in no significant difference between the 2 groups.^[4]^ In contrast to the results of Higuchi et al, the present study showed that 24 (52.17%) patients in the CwG group were positive for rotavirus, while only 6 (17.14%) patients in the FS group were positive for rotavirus, resulting in a significant difference between the 2 groups (*P* < .01),which suggested that the rate of rotavirus infection in children with CwG was higher than that in children with FS. Our current study also showed that FS associated with mild gastroenteritis was more likely to relapse than CwG in long-term follow-up, and 5 patients with FS eventually developed into epilepsy. However, none of patients with CWG eventually developed into epilepsy in our study. This indicated that FS had a worse prognosis, which has been confirmed by previous researches. Previous studies reported that none of patients with CWG developed epilepsy, while about 3% of patients with FS developed epilepsy after long-term follow-up.^[24,25]^ Previous studies reported that the risk of future epilepsy in children with complex febrile seizures was around 4% to 6%, and children with simple febrile seizures was around 1%.^[26]^

Although CwG and FS associated with mild gastroenteritis can be preliminarily identified based on clinical symptoms, the diagnosis is very subjective and uncertain. Therefore, it is particularly important to search for objective laboratory indicators.

The S100B protein is a calcium binding protein and is specifically present in the nervous system. It is mainly secreted by glial cells in the central nervous system and is a marker of glial cell injury. When the integrity of glial cells is damaged, S100B can enter the extracellular fluid and then enter the blood through the blood brain barrier. It can be used for diagnosis and evaluation of neuropsychiatric diseases such as schizophrenia, mood disorders, cerebral palsy and traumatic brain injury.^[27,28]^ This study retrospectively analyzed serum S100B protein expression in 46 patients in the CwG group and compared the results to those of 35 patients in FS group. No significant difference was identified, suggesting that S100B protein is not ideal for the differential diagnosis between CwG and FS associated with mild gastroenteritis.

NSE is another brain injury marker. It is an enzyme that forms a dimer with a molecular weight of 78 kDa and a half-life of 24 hours. It is specifically present in neurons and neuroendocrine cells and is a marker of neuronal damage.^[27]^ When neurons exhibit edema, degeneration and necrosis, NSE is released into the blood through the damaged cell membrane and blood-brain barrier, and the blood level of NSE is positively correlated with the degree of brain injury.^[29]^ NSE can be used for the diagnosis and assessment of severity of brain injuries, such as brain tumors, traumatic brain injury, epilepsy, cerebral infarction, intracranial hemorrhage, Guillain Barre syndrome and Alzheimers disease.^[28]^ Previous study have shown that the elevated levels of NSE may be beneficial for the identification and diagnosis of epileptic seizures clinically.^[30]^ Another study of meta-analysis reported random-effects meta-analysis of 26 studies, including 1360 patients with epilepsy, and 1256 healthy control, revealed that childhood epilepsy exhibited meaningfully increased CSF and serum levels of NSE compared with controls. The results demonstrated that childhood epilepsy exhibits significantly elevated levels of NSE in the CSF and serum, thus strengthening the association between increased NSE levels and epilepsy.^[31]^ CwG is clinically partially similar to epilepsy, so NSE may be beneficial for the identification and diagnosis of CwG clinically. The present study found that the serum NSE levels of the CwG group were higher than those of the FS group (14.046 (11.095, 19.266) pg/ml and 9.034 (7.158, 12.165) pg/ml, respectively). To further understand the correlation between NSE and the number of convulsions or the duration of convulsions, correlation analysis was performed, which found no correlation between the serum NSE level and the number of convulsions in the both CwG and FS groups (*P* > .05). Similarly, the serum NSE level was not correlated the total duration of convulsions in the CwG group or the FS group (*P* > .05), which is consistent with previous reports.^[32]^ This may be related to the short duration of each convulsion in patients in both groups. None of the subjects in this study had a convulsion that lasted longer than 30 minutes. Studies have shown that tonic-clonic seizures lasting longer than 30 minutes or complicated partial seizures lasting longer than 60 minutes may cause neuronal injury.^[33]^ Besides, both in adults and children, etiology and age are more relevant factors than seizure duration.^[34]^

The relevant mechanisms responsible for the elevated NSE levels in the CwG group are still unclear. It is speculated that this phenomenon may be associated with brain damage caused by gastroenteritis-associated virus-induced brain infection or may also be related to the release of enterotoxins, amines, peptides, cytokines, prostaglandins and nitrous oxide, which eventually cause to neurotoxicity and further lead to brain damage.^[7]^ However, We suspected that the increase of NSE in CwG was only temporary, because the overall prognosis of CwG was good and the brain injury was reversible. Secondly, normal levels of NSE do not predict a good prognosis for neurological disorders. For example, Ko FJ et al reported that a patient died of Reye syndrome did not show a very high level of NSE in the serum or CSF.^[35]^ Suzuki Y et al reported that there were no significant differences in serum NSE levels between the group with west syndrome (n = 18) and infants with normal neurologic development (n = 28), And the serum NSE value in the group with an acute neurologic insult (n = 10) was significantly higher than that for the west syndrome.^[36]^ However, west syndrome may has a poor prognosis.^[37]^ Therefore, when different diseases have different levels of NSE, NSE does not fully predict the prognosis, and the prognosis of the disease is related to the characteristics of the disease itself. Finally, there may be another hypothesis. CwG has a good prognosis, suggesting that brain injury is not serious, and the elevation of NSE may not be entirely derived from brain tissue. Previous studies showed that there were a large number of nerve cells, enteroglia and neuroendocrine cells in the intestinal wall, as well as a large number of NSE.^[38,39]^ In China, previous study showed that intestines of patients with CWG had fewer probiotics than patients with mild gastroenteritis, and the function of the intestinal mucosal barrier was worse in patients with CWG than in patients with mild gastroenteritis.^[40]^ Another study showed that serum nitric oxide in patients with CWG were significantly higher than patients with FS. Excessive nitric oxide and its metabolites led to intestinal mucosal damage and intestinal barrier dysfunction.^[41]^ The damage of the intestinal wall further led to the elevation of NSE. Therefore, patients with CwG had higher NSE, and the elevation of NSE did not represent serious brain damage, and could not be used to judge the prognosis. However, this needs to be confirmed by further experiments. Although NSE level do not predict prognosis for different diseases, this do not affect the diagnostic value of NSE in CwG in the acute phase.

To further understand the diagnostic value of NSE, the serum NSE level was used as a predictor to determine whether the study subjects could be diagnosed with CwG, and a ROC curve was generated. The results showed that the AUC of NSE was 0.806, *P* = .000, which was statistically significant, indicating that the NSE level had value for predicting diagnosis of CwG. The Youden index was largest (0.605) with a serum NSE level of 10.460 pg/ml, and the sensitivity and specificity when this value was used for the prediction of a CwG diagnosis were 89% and 71%, respectively. The protein levels of serum NSE were detected by chemiluminescence. Each test costs $11.50 and takes about 2 hours in our hospital. Therefore, detection of NSE has a strong feasibility in emergency department. However, not all patients with seizures associated with mild gastroenteritis need to be tested for NSE. The detection of NSE is a good choice only when the clinical diagnosis is unclear.

This study has some limitations. First of all, because this is a retrospective study, we did not conduct dynamic monitoring of NSE, including at onset, post-convulsion, and recovery, in patients in the CwG or FS groups. Prospective studies are required for further clarification. Secondly, the exact mechanism of elevation of NSE in patients with CWG is still unknown, which needs to be further elucidated. Finally, It is still unknown whether patients with MRI abnormalities have higher levels of NSE, and because our hospital and our research institution did not have the techniques for detecting NSE of cerebrospinal fluid, we did not test it in our current study, which will be further confirmed in future studies.

In summary, CwG and FS associated with mild gastroenteritis are 2 different diseases in the spectrum of seizures associated with mild gastroenteritis. FS associated with mild gastroenteritis is more likely to relapse than CwG in long-term follow-up, and FS is more likely to develop into epilepsy. Although they both have unique characteristics and clinical symptoms, much subjectivity and uncertainty exists in terms of symptom-only identification. The combination of the serum NSE level, as an objective indicator, and clinical symptoms may assist in the differential diagnosis between CwG and FS associated with mild gastroenteritis. However, the exact mechanism of elevation of NSE in patients with CwG is still unknown, which needs to be further elucidated and confirmed. Meanwhile, it needs to be further verified clinically through more clinical cases.

## Acknowledgments

The authors thank the staff of the Department of Neurology of Children's Hospital of Jiangxi Province for their valuable efforts.

## Author contributions

**Conceptualization:** Hui Chen, Yong Chen, Jianmin Zhong.

**Data curation:** Hui Chen, Yong Chen.

**Formal analysis:** Hui Chen, Yong Chen.

**Investigation:** Hui Chen, Yong Chen, Jianmin Zhong.

**Methodology:** Hui Chen, Yong Chen, Jianmin Zhong.

**Project administration:** Yong Chen, Jianmin Zhong.

**Resources:** Hui Chen, Yong Chen.

**Software:** Yong Chen.

**Supervision:** Jianmin Zhong.

**Validation:** Jianmin Zhong.

**Visualization:** Hui Chen, Yong Chen.

**Writing – original draft:** Hui Chen.

**Writing – review & editing:** Hui Chen, Yong Chen, Jianmin Zhong.
